# Complex Networks Approach for Analyzing the Correlation of Traditional Chinese Medicine Syndrome Evolvement and Cardiovascular Events in Patients with Stable Coronary Heart Disease

**DOI:** 10.1155/2015/824850

**Published:** 2015-03-03

**Authors:** Zhuye Gao, Siwei Li, Qinghua Shang, Yang Jiao, Xuezhong Zhou, Changgeng Fu, Hao Xu, Dazhuo Shi, Keji Chen

**Affiliations:** ^1^China Heart Institute of Chinese Medicine, China Academy of Chinese Medical Sciences, Beijing 100091, China; ^2^Beijing University of Chinese Medicine, Beijing 100029, China; ^3^School of Computer and Information Technology and Beijing Key Lab of Traffic Data Analysis and Mining, Beijing Jiaotong University, Beijing 100044, China

## Abstract

This is a multicenter prospective cohort study to analyze the correlation of traditional Chinese medicine (TCM) syndrome evolvement and cardiovascular events in patients with stable coronary heart disease (CHD). The impact of syndrome evolvement on cardiovascular events during the 6-month and 12-month follow-up was analyzed using complex networks approach. Results of verification using Chi-square test showed that the occurrence of cardiovascular events was positively correlated with syndrome evolvement when it evolved from toxic syndrome to Qi deficiency, blood stasis, or sustained toxic syndrome, when it evolved from Qi deficiency to blood stasis, toxic syndrome, or sustained Qi deficiency, and when it evolved from blood stasis to Qi deficiency. Blood stasis, Qi deficiency, and toxic syndrome are important syndrome factors for stable CHD. There are positive correlations between cardiovascular events and syndrome evolution from toxic syndrome to Qi deficiency or blood stasis, from Qi deficiency to blood stasis, or toxic syndrome and from blood stasis to Qi deficiency. These results indicate that stable CHD patients with pathogenesis of toxin consuming Qi, toxin leading to blood stasis, and mutual transformation of Qi deficiency and blood stasis are prone to recurrent cardiovascular events.

## 1. Introduction

China and some other developing countries are experiencing an increase in prevalence of cardiovascular diseases (CVDs), which is now the leading cause of mortality and morbidity [1, 2]. According to the new concept, the stable coronary heart disease (CHD) includes chronic stable angina pectoris, old myocardial infarction (OMI), and successful postrevascularization without symptom [3, 4]. In recent years, accumulating evidences have demonstrated that the interventions with TCM for stable CHD patients reduced the incidence of acute cardiovascular events [5, 6]. In Chinese medicine (CM), syndrome is a combination of medical problems that commonly go together, which may show the existence of a particular disease condition [7]. CHD is thought to be a result of “deficiency in origin and excess in superficiality.” Deficiency in origin includes deficiency in* Qi*, blood,* yin,* and* yang*; excess in superficiality includes* Qi *stagnation, blood stasis, phlegm, and cold coagulation. Among these pathogenic factors, blood stasis is generally considered as an essential syndrome and a key etiology throughout the process of CHD [8, 9]. However, why do patients with the same blood stasis syndrome have different prognosis? That is, why do some patients stay at stable condition in the long term while others develop cardiovascular events? We have come up with a hypothesis that the complicated interactions among TCM syndromes play a significant role in the progression of CHD. Complex networks coming from graph analysis are able to demonstrate correlation and quantify the analyses of functional connectivity network by a set of values. Therefore, to explore the correlation between TCM syndrome evolvement and cardiovascular events on stable CHD patients, we conducted a cohort study using complex networks approach, in order to provide some valuable evidences for establishing a more effective intervention strategy with CM to stable CHD.

## 2. Materials and Methods

### 2.1. Patients

1503 cases with stable CHD were recruited from 5 hospitals from October 2007 to July 2010 in China. The research followed guidelines of the Declaration of Helsinki for humans and was approved by the institutional human experimentation committee. The informed consent was also obtained. The source of patients was shown in Table 1.

### 2.2. Diagnostic Criteria

Coronary angiography showed stenosis ≥50% in at least one coronary artery or previous myocardial infarction. Classification of CHD syndrome referred to the “Criterion of Syndrome Differentiation for CHD” by Cardiovascular Specialty Committee, China Association of Integrative Medicine [10]. Toxin syndrome differentiation was made according to the “Diagnostic Criterion of Toxin Syndrome in Stable CHD” [11].

### 2.3. Inclusion and Exclusion Criteria

Inclusion criteria included (1) asymptomatic, stable exertional angina patients or postrevascularization patients with stable condition, (2) age ≤ 80, and (3) signed informed consent.

Exclusion criteria included (1) ACS, (2) revascularization treatment within 1 month, (3) acute infectious diseases, (4) severe heart failure or ejection fraction (EF) < 35%, (5) complication with severe valvular disease or cardiomyopathy, (6) complication with pulmonary heart disease or respiratory failure, (7) renal insufficiency, serum creatinine (Cr) > 221 *μ*mol/L for male or >177 *μ*mol/L for female, (8) liver dysfunction, alanine aminotransferase (ALT) more than 3 times higher than upper reference range limit, or complication with liver cirrhosis, (9) severe hematopoietic system diseases, (10) severe mental illness, (11) malignant tumor, and (12) organ transplant receivers.

### 2.4. Clinical Design and Data-Collecting Methods

This prospective cohort study investigated and collected the clinical information of patients with stable CHD, based on the information from the CHD Clinical Research Information Sharing System (CM structured electronic medical record system as the core medical business platform and for transferring and storing multimedia data of different research centers through virtual network transmission methods).

### 2.5. Procedures

(1) There is formulation of case report form (CRF). (2) Researchers selection: selective graduate students with professional medical knowledge and clinical experience were trained with research methods, techniques, and content of investigation, and they become researchers after passing certain examinations after the training. (3) Preinvestigation: find out the deficiency of the research method and optimize the questionnaire according to a pilot study. (4) Information acquisition: patients' condition was recorded, including physical characteristics, personal medical conditions and medications, personal and family medical history, current symptoms, tongue manifestation, pulse condition, and syndrome differentiation from direct observation of looking, listening, questioning, and pulse-feeling during face-to-face interview in natural light under quiet circumstance. The syndrome differentiation was confirmed by two integrative cardiologists (at least associate chief physician). (5) Follow-up: the recurrent cardiovascular events were followed up at 6 months and 12 months. Cardiovascular events were defined as nonfatal myocardial infarction, stroke, revascularization (including percutaneous coronary intervention (PCI) and/or coronary artery bypass grafting (CABG)), and cardiac death. (6) Quality control: in each survey point, integrative cardiologists (at least associate chief physician) were designated as guide for quality control to ensure the authenticity and accuracy of the investigation records. (7) Data management: the collected data was managed by Mysql Server 5.0 and adopted by double data-entry and verification to ensure that the information was accurate and reliable. To analyze the impact of CM syndrome evolution on cardiovascular events, only the data of patients who had completed the follow-up was analyzed.

### 2.6. Statistical Analysis

Statistical software SPSS 13.0 was used for the analysis. Continuous variables were expressed as mean ± standard deviation. Categorical data were described by frequency tables, percentage, or constituent ratio and analyzed by Chi-square test. The impact of syndrome evolvement on cardiovascular events was modeled by complex networks with multiscale backbone based network comparison algorithm and validated by Chi-square test [12]. Statistical significance was set at *P* value <0.05.

## 3. Results

### 3.1. Demographics and Clinical Characteristics

1503 stable CHD patients were enrolled. Cardiovascular events occurred in 35 cases within 6 months, and 37 cases had cardiovascular events during 6 to 12 months after the study began. Among those patients, 5 (0.33%) had cardiac death, 7 (0.47%) had nonfatal myocardial infarction, 11 (0.73%) had stroke, and 49 (3.26%) had coronary revascularization. Totally 1,333 of 1,503 stable CHD patients met the inclusion criteria of complex networks model. Among them, 959 (71.9%) cases were males and 374 (28.1%) cases were females. The average age in the selected patients was 61.72 ± 9.30 years (range 26–80).

### 3.2. Analysis Model on Complex Networks

**Figure 1:**
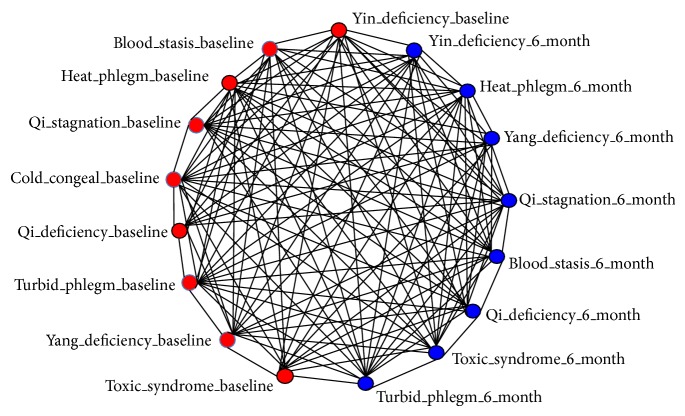
Wiring diagrams for the complex networks obtained from clinical data of recurrent cardiovascular events (node degree = 0). Notes: Blood_stasis_baseline represents blood stasis at the baseline and Blood_stasis_6_month represents blood stasis at the 6-month follow-up (the same as in Figure 2). In the network, the nodes of the link present the corresponding syndrome, the links present that at least one patient suffers the syndrome, and the weight of the links equals the number of patients. For example, the weight of link between Blood_stasis_baseline and Qi_deficiency_6_month was 26, which represents the fact that 26 of 30 cases with blood stasis at the baseline transformed to Qi deficiency at the 6-month follow-up.

**Figure 2:**
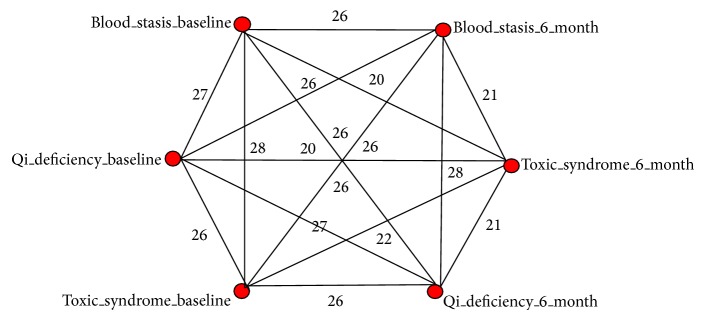
Wiring diagrams for the complex networks obtained from clinical data of recurrent cardiovascular events (node degree = 17).

Figure 1 shows the relationship among the syndromes with recurrent cardiovascular events.  Figure 2 shows the diagram of the best predictive complex networks model obtained when the node degree was seventeen. The diagram implicates pivotally 6 nodes, including blood stasis at the baseline, Qi deficiency at the baseline, toxic syndrome at the baseline, Qi deficiency at the 6-month follow-up, blood stasis at the 6-month follow-up, and toxic syndrome at the 6-month follow-up. Using Chi-square test to verify the results of complex networks, it shows that the occurrence of cardiovascular events was positively correlated with syndrome evolvement when it evolved from toxic syndrome at the baseline to Qi deficiency at the 6-month follow-up, blood stasis at the 6-month follow-up, or sustained toxic syndrome at the 6-month follow-up, when it evolved from Qi deficiency at the baseline to blood stasis at the 6-month follow-up, toxic syndrome at the 6-month follow-up, or sustained Qi deficiency at the 6-month follow-up, and when it evolved from blood stasis at the baseline to Qi deficiency at the 6-month follow-up (*P* < 0.05 for all). It was not statistically significant when it evolved from blood stasis to toxic syndrome at the 6-month follow-up or sustained blood stasis at the 6-month follow-up (*P* = 0.089, 0.852) (Table 2).

## 4. Discussion

In this paper, we conducted a one-year follow-up multicenter prospective cohort study, which investigated and collected the clinical information of patients with stable CHD including the syndrome type and the recurrent cardiovascular events. We detected and discovered the correlation between syndrome evolvement and recurrent cardiovascular events with stable CHD in the way of complex networks. The results indicate that blood stasis, toxic syndrome, and Qi deficiency are important syndrome factors and there exists strong correlation between syndrome evolvement and recurrent cardiovascular events in stable CHD. Recurrent cardiovascular events are prone to happen when it evolved from toxic syndrome to Qi deficiency, blood stasis, or sustained toxic syndrome, when it evolved from Qi deficiency to blood stasis, toxic syndrome, or sustained Qi deficiency, and when it evolved from blood stasis to Qi deficiency, while evolving from blood stasis to toxic syndrome or sustained blood stasis did not affect the rate of recurrent cardiovascular events. In other words, patients who had blood stasis at the baseline and continued to have blood stasis without any syndrome evolvement at 6 months were not prone to have cardiovascular events. These suggest that toxin and blood stasis consuming healthy Qi play a pivotal role in the process of cardiovascular events in stable CHD patients. Combining the theory of TCM, these also indicate that the syndromes evolvement discipline of mutual transformation of blood stasis, toxin, and Qi deficiency might be the underlying pathogenesis of the recurrent cardiovascular events.

Complex network is a method and perspective to study complexity of the system, and it is the basic framework of a complex system [13]. Network representation has been natural in abstraction of many complex systems. The complexity of a network structure can be characterized by the connectivity properties of the interaction pathways (links) between network components (nodes). The degree of a node is the number of its links connected to other nodes. Complex network science has witnessed many developments in the last decade since many real-world networks were found to have a variety of topological structures. Complex network is very helpful to portray a complex system. Complex network research has been involved in various fields, like ecology, sociology, physics, medicine, and biology (such as cellular networks, the role of protein-protein networks) [14–18]. In recent years, network pharmacology based on complex network, the approach to explain the human disease and curative mechanism, is a major breakthrough in medical research [16]. Meanwhile, complex network analysis method has been showed to be a promising approach to discover core syndrome and the regularities of syndrome evolvement in the disease progression [19].

TCM is a discipline with its own distinct methodologies and philosophical principles. Typically, a number of herbs are combined to form a formula and different formulae are prescribed for different syndromes. In this study, we verified and confirmed the positive correlation between syndrome evolvement and recurrent cardiovascular events by using complex network method and the Chi-square test. Interesting regularities are discovered and the results are consistent with our previous results based on multifactor dimensionality reduction (MDR) approach [20]. Also these results contain some specific knowledge held in the diagram of complex network, which demonstrate the number of cases in detail. For example, the weight of link was 26 between Blood_stasis_baseline and Qi_deficiency_6_month, which represents 26 of 30 cases with blood stasis at the baseline transformed to Qi deficiency at the 6-month follow-up. This could be used to further investigate the intervention with TCM or novel drug development.

Integrative medicine is a kind of complex interventions. It is difficult for this method to totally adapt to the clinical features of CM and integrative medicine as complex interventions. As a result, one possible way to solve this issue is to improve and integrate with the existing method and to utilize the evaluation model on complex interventions from abroad. The application of data mining in the therapeutic evaluation of integrative medicine has broad prospects [21]. Due to the complexity in TCM clinical operations, complex network provides a promising approach to investigate the complicated structures such as syndrome evolvement relationships hidden in the process of disease and to elucidate the holistic and nonlinear and dynamics characteristics of TCM to some extent. Based on the complicated and multisource data of coronary heart disease clinical diagnosis and treatment, analysis of syndrome evolution's influence on cardiovascular events and presentation in the form of chart will help to summarize the complicated characteristics of syndrome evolution and to explore the key pathogenesis of stable CHD [22].

We have proposed and verified a hypothesis of “blood stasis and toxin” considering blood stasis was a constant pathogenesis in CHD, while “toxin” was the trigger for transforming to recurrent cardiovascular events [23]. However, we should fully recognize that blood stasis is the important pathological factor but not the only one. In the context of emphasizing the crucial procedure of syndrome and comprehensively considering impact of toxin syndrome in the syndrome evolvement with stable CHD, early identification and intervention of toxin, blood stasis, and Qi deficiency according to the syndrome evolvement regularities of stable CHD will be able to make the most of the CM advantage of “preventive treatment of disease” and thus are of great significance for the secondary prevention of CHD and further reduce the recurrent cardiovascular events.

## Figures and Tables

**Table 1:** Source of patients.

Hospital	Case (male/female)	Percentage of total
Xiyuan Hospital affiliated to China Academy of Chinese Medical Science	198 (142/56)	13.17%
China-Japan Friendship Hospital	461 (335/126)	30.67%
Anzhen Hospital affiliated to Capital University of Medical Sciences	493 (359/134)	32.80%
Tongren Hospital affiliated to Capital University of Medical Sciences	251 (162/89)	16.70%
Fujian Hospital of Integrated Chinese and Western Medicine	100 (67/33)	6.65%

**Table 2:** Correlation verification of the complex network analysis results [case (%)].

Syndrome evolvement	The occurrence of cardiovascular events after 6 months			*χ* ^2^	*P*
		No	Yes		
Qi_deficiency_baseline to Toxic_syndrome_6_month	No	905 (69.8)	17 (45.9)	9.623	0.003
	Yes	391 (30.2)	20 (54.1)		

Qi_deficiency_baseline to Blood_stasis_6_month	No	612 (47.2)	11 (29.7)	4.422	0.044
	Yes	684 (52.8)	26 (70.3)		

Qi_deficiency_baseline to Qi_deficiency_6_month	No	741 (57.2)	10 (27.0)	13.293	<0.001
	Yes	555 (42.8)	27 (73.0)		

Blood_stasis_baseline to Toxic_syndrome_6_month	No	782 (60.3)	17 (45.9)	3.104	0.089
	Yes	514 (39.7)	20 (54.1)		

Blood_stasis_baseline to Blood_stasis_6_month	No	358 (27.6)	11 (29.7)	0.08	0.852
	Yes	938 (72.4)	26 (70.3)		

Blood_stasis_baseline to Qi_deficiency_6_month	No	651 (50.2)	11 (29.7)	6.048	0.019
	Yes	645 (49.8)	26 (70.3)		

Toxic_syndrome_baseline to Toxic_syndrome_6_month	No	909 (70.1)	15 (40.5)	14.818	<0.001
	Yes	387 (29.9)	22 (59.5)		

Toxic_syndrome_baseline to Blood_stasis_6_month	No	759 (58.6)	11 (29.7)	12.26	0.001
	Yes	537 (41.4)	26 (70.3)		

Toxic_syndrome_baseline to Qi_deficiency_6_month	No	902 (69.6)	11 (29.7)	26.496	<0.001
	Yes	394 (30.4)	26 (70.3)		
